# Distinct Biological Potential of *Streptococcus gordonii* and *Streptococcus sanguinis* Revealed by Comparative Genome Analysis

**DOI:** 10.1038/s41598-017-02399-4

**Published:** 2017-06-07

**Authors:** Wenning Zheng, Mui Fern Tan, Lesley A. Old, Ian C. Paterson, Nicholas S. Jakubovics, Siew Woh Choo

**Affiliations:** 1 Department of Biological Sciences, Xi’an Jiaotong-Liverpool University, Suzhou Dushu Lake Science and Education Innovation District, Suzhou Industrial Park, 215123 Suzhou, P. R. China; 2 Department of Oral and Craniofacial Sciences, Faculty of Dentistry, University of Malaya, 50603 Kuala Lumpur, Malaysia; 3 Oral Cancer Research and Coordinating Centre, Faculty of Dentistry, University of Malaya, 50603 Kuala Lumpur, Malaysia; 4 Centre for Oral Health Research, School of Dental Sciences, Newcastle University, Newcastle upon Tyne, NE2 4BW United Kingdom; 5 Genome Solutions Sdn Bhd, Suite 8, Innovation Incubator UM, Level 5, Research Management & Innovation Complex, University of Malaya, 50603 Kuala Lumpur, Malaysia

## Abstract

*Streptococcus gordonii* and *Streptococcus sanguinis* are pioneer colonizers of dental plaque and important agents of bacterial infective endocarditis (IE). To gain a greater understanding of these two closely related species, we performed comparative analyses on 14 new *S*. *gordonii* and 5 *S*. *sanguinis* strains using various bioinformatics approaches. We revealed *S*. *gordonii* and *S*. *sanguinis* harbor open pan-genomes and share generally high sequence homology and number of core genes including virulence genes. However, we observed subtle differences in genomic islands and prophages between the species. Comparative pathogenomics analysis identified *S*. *sanguinis* strains have genes encoding IgA proteases, mitogenic factor deoxyribonucleases, nickel/cobalt uptake and cobalamin biosynthesis. On the contrary, genomic islands of *S*. *gordonii* strains contain additional copies of *comCDE* quorum-sensing system components involved in genetic competence. Two distinct polysaccharide locus architectures were identified, one of which was exclusively present in *S*. *gordonii* strains. The first evidence of genes encoding the CylA and CylB system by the α-haemolytic *S*. *gordonii* is presented. This study provides new insights into the genetic distinctions between *S*. *gordonii* and *S*. *sanguinis*, which yields understanding of tooth surfaces colonization and contributions to dental plaque formation, as well as their potential roles in the pathogenesis of IE.

## Introduction

Oral streptococci including *Streptococcus gordonii* (Sg) and *Streptococcus sanguinis* (Ss), are among the most common colonizers of oral biofilms on tooth surfaces, known as dental plaque^1^. Sg and Ss have the capacity to attach to components of the salivary pellicle, as well as to other oral bacteria through a broad range of adhesin proteins that are expressed on the cell surface^2^. These interactions are thought to be instrumental in the initiation and progression of dental plaque formation. In addition, Sg and Ss are able to invade the bloodstream and are important causative agents of the rare, but life-threatening disease bacterial infective endocarditis (IE)^3^. These α-hemolytic oral streptococci have also been identified recently in neutropaenic bloodstream infections^4^.

Originally, Sg and Ss were considered to be the same species, ‘*S*. *sanguis*’. Despite sharing approximately 97% sequence identity across the 16S rRNA gene, they were recognized as distinct species in 1989, on the basis of biochemical, physiological and serological profiles^5^. In particular, almost all Sg strains utilize amygdalin, produce α-D-glucosidase, β-mannosidase and α-L-fucosidase, and have strong alkaline phosphatase activity, whereas these characteristics are rare in Ss. On the other hand, Ss strains produce IgA protease, which is not present in Sg. More recent phylogenomic analysis indicates that Sg and Ss form a group that is separate from other streptococci but is closely related to the ‘*S*. *sinensis*’ clade containing the species *S*. *oligofermentans*, *S*. *sinensis* and *S*. *cristatus* ^6^. Within the mouth, Sg and Ss appear to share the same habitat, and are both found predominantly on tooth surfaces, either above or below the gumline^7^. There is some evidence that the two species may play distinct roles in oral health and disease. For example, increased levels of Ss, but not Sg, have been associated with periodontal health^8, 9^. The associations with dental caries are more complex, and different studies have shown different associations. Nevertheless, there appear to be some differences between levels of Ss and Sg in health versus disease^1^. For example, Ss is almost always found more frequently or in higher numbers than Sg^7^. It is not clear how Sg survives as a species, even though it is apparently not as well adapted for growth in dental plaque as Ss. One possible explanation is that Sg has an extensive battery of cell surface adhesins that recognize a wide range of substrates and enable Sg to adhere to different surfaces. For example, the Sg cell surface adhesin Hsa mediates strong binding to salivary pellicle, and enables Sg to outcompete Ss for adhesion to pellicle-coated surfaces *in vitro* ^10^.

This study aimed to identify and compare the core and pan genomes of Ss and Sg in order to enhance our understanding of the differences between these two closely-related species. Comparative genome analyses were performed in order to investigate potential differences in phylogeny, virulence, biology and genomics.

## Results and Discussion

### Genome Overview

Fourteen strains of Sg and 5 strains of Ss were successfully sequenced, assembled, annotated and identified. The assembled genomes have an average genomic size of 2,290,927 bp with an average G + C content of 41.2%. The genome completeness and identities of the 14 Sg strains ranged from 88–95% and 95–98%, respectively. The five Ss strains achieved genome completeness between 84% to 97% and genome identities between 95 to 96%. The Rapid Annotation Subsystem Technology (RAST) annotation pipeline predicted approximately 2,117 to 2,429 protein-coding genes and 2–6 ribosomal RNA genes in both *Streptococcus* species. Sg genomes harbor between 38–47 transfer RNAs with an average GC content of 40.5%, whereas Ss genomes have 40–49 transfer RNAs with a relatively higher average GC content of 43.2% (Table 1).

**Table 1:** Summary of the genome features of 19 newly sequenced *Streptococcu*s strains.

Strain	PV40	Blackburn	Channon	FSS2	FSS3	
Status of genome	Contigs	Contigs	Contigs	Contigs	Contigs	
Genome Size (Mbp)	2.19	2.16	2.23	2.19	2.31	
GC content (%)	40.5	40.5	40.6	40.5	40.2	
Number of CDS	2170	2132	2236	2165	2212	
Number of tRNAs	46	42	42	46	42	
Number of rRNAs	3	3	3	3	5	
Genome Identity (%)	98	96	96	98	96	
Genome Mapped (%)	95	90	89	92	92	
Number of final contigs	43	50	33	18	382	
**Strain**	**FSS8**	**M5**	**M99**	**MB666**	**MW10**	
Status of genome	Contigs	Contigs	Contigs	Contigs	Contigs	
Genome Size (Mbp)	2.15	2.16	2.17	2.31	2.19	
GC content (%)	40.6	40.6	40.5	40.3	40.5	
Number of CDS	2132	2117	2128	2314	2158	
Number of tRNAs	38	41	43	46	38	
Number of rRNAs	2	3	3	3	3	
Genome Identity (%)	95	95	95	96	98	
Genome Mapped (%)	90	88	89	90	92	
Number of final contigs	41	67	45	20	27	
**Strain**	**NCTC 7863**	**FSS4**	**FSS9**	**MB451**	**PJM8**	
Status of genome	Contigs	Contigs	Contigs	Contigs	Contigs	
Genome Size (Mbp)	2.3	2.31	2.43	2.45	2.37	
GC content (%)	43.3	43.2	43.1	42.9	43.2	
Number of CDS	2284	2294	2418	2429	2326	
Number of tRNAs	40	49	47	47	42	
Number of rRNAs	6	3	3	3	5	
Genome Identity (%)	95	95	95	96	95	
Genome Mapped (%)	84	85	97	94	92	
Number of final contigs	110	63	20	27	162	
**Strain**	**PK488**	**SK12**	**SK120**	**SK184**	*Sg Challis	*Ss SK36
Status of genome	Contigs	Contigs	Contigs	Contigs	Complete	Complete
Genome Size (Mbp)	2.2	2.15	2.16	2.26	2.2	2.39
GC content (%)	40.4	40.6	40.4	40.5	40.5	43.4
Number of CDS	2176	2143	2119	2273	2173	2385
Number of tRNAs	37	47	47	42	59	61
Number of rRNAs	3	3	3	3	12	12
Genome Identity (%)	96	95	96	97	100	100
Genome Mapped (%)	91	89	90	92	100	100
Number of final contigs	46	27	28	53		

The details include genome size, GC content (%), number of coding sequences (CDSs), tRNAs, rRNA, genome identity and the percentage of the genome mapped to the reference. There are a total of 14 Sg strains and 5 Ss strains (underlined). The reference genomes of Sg Challis and Ss SK36 are marked with asterisks.

### Phylogenetic inference

**Figure 1:**
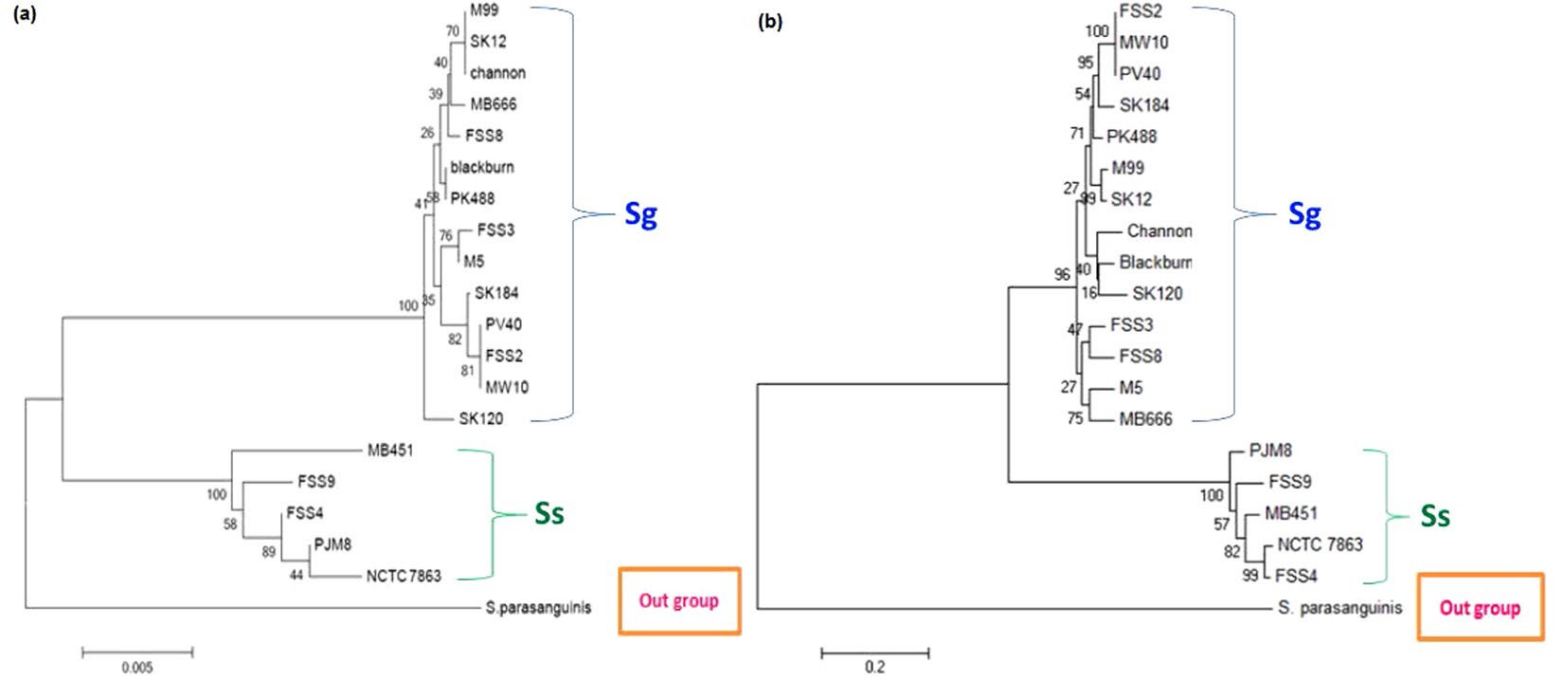
The single gene marker 16S rRNA phylogenetic tree (**a**) and core genome-SNP phylogenetic tree (**b**) classified 14 strains of Sg and 5 strains of Ss into different species clades using *Streptococcus parasanguinis* as the outgroup.

To identify the taxonomic position of each sequenced isolate, we constructed phylogenetic trees using both single gene and whole-genome approaches. The single gene approach utilized the 16S rRNA housekeeping gene to construct a phylogenetic tree using *Streptococcus parasanguinis* as an outgroup species (Fig. 1a). 16S rRNA gene sequences have been widely used as gene markers to differentiate species of *Streptococcus* genus particularly for α-hemolytic streptococci including Ss and Sg^11^. Our 16S rRNA-based phylogenetic tree clearly classified the 19 *Streptococcus* strains into two clades: 14 strains of Sg (PV40, Blackburn, Channon, FSS2, FSS3, FSS8, M5, M99, MB666, MW10, PK488, SK12, SK120 and SK184) and 5 strains of Ss (NCTC 7863, FSS4, FSS9, MB451 and PJM8). Sg and Ss are closely related and are approximately 97% identical across the 16S rRNA gene.

To further support our classification results, we reconstructed a phylogenetic tree using core-genome Single Nucleotide Polymorphism (SNP) data, providing a more robust tree compared to the single gene approach. Encouragingly, our data showed that the classification results from the core-genome SNP-based tree (Fig. 1b) were consistent with the classification from the 16S rRNA-based tree with only a few slight differences. For example, our 16S rRNA-based phylogenetic tree (Fig. 1a) classified Sg FSS8 and Sg MB666 in the same clade while Sg FSS3 and Sg M5 were in a separate clade. On the other hand, Sg M5 and Sg MB666 are grouped under the same clade whereas Sg FSS8 and Sg FSS3 are housed under their adjacent divided clade in the core-genome SNP-based tree (Fig. 1b). Interestingly, Sg FSS2, MW10 and PV40 are almost identical at the level of 16S rRNA gene sequence and whole genome SNP, even though these strains were isolated from different sources at different times. Sg FSS2 and PV40 were from Newcastle upon Tyne, UK, Sg MW10 was isolated in Sydney Australia; Sg PV40 and MW10 were from the oral cavity, whereas FSS2 originated from a case of bacterial infective endocarditis.

### Sg and Ss have open pan-genomes

**Figure 2:**
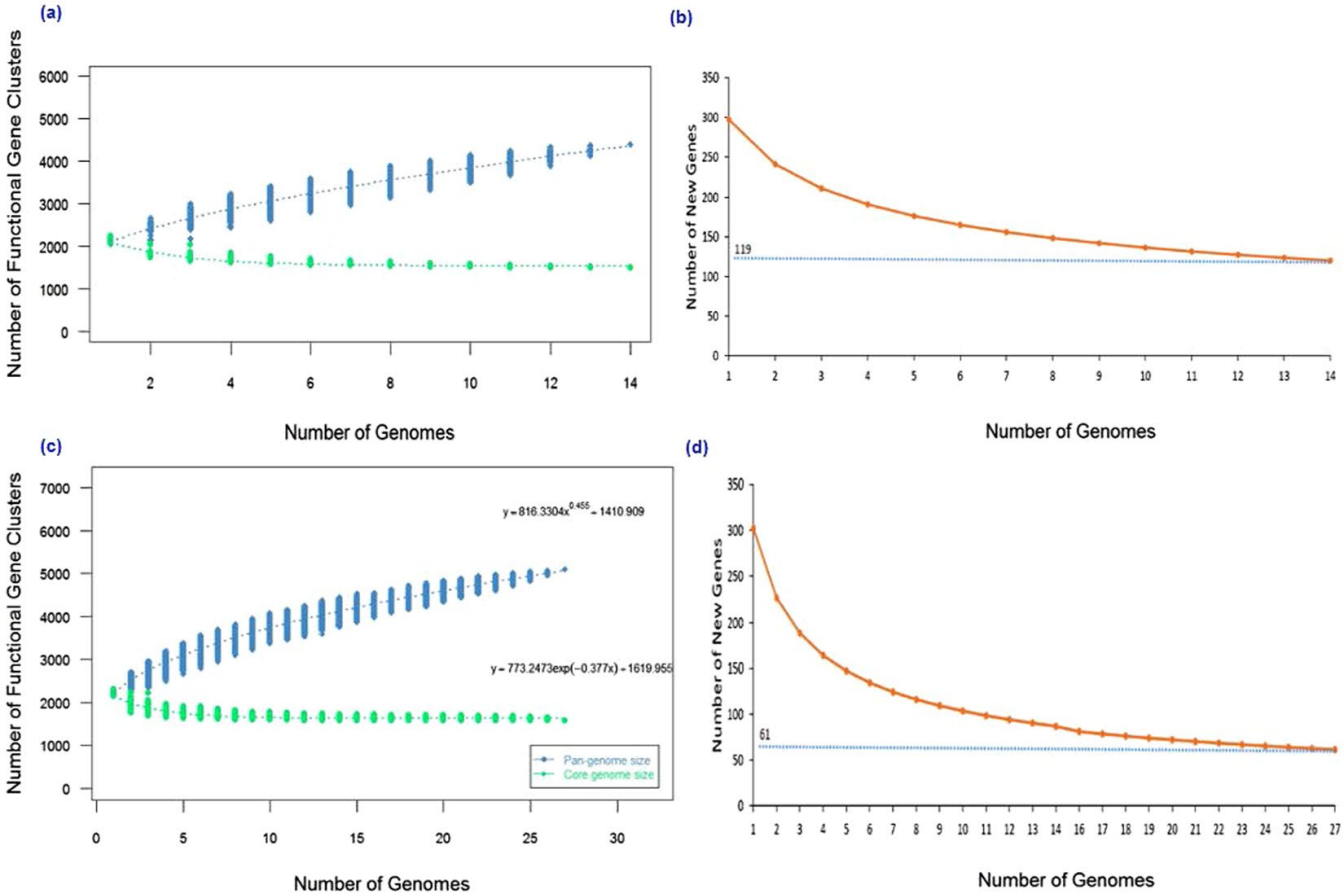
Pan-genome analyses. Curves for Sg (**a**) and Ss (**c**) pan-genomes and core genomes. The blue dots denote the *Streptococcus* pan-genome size for each genome comparison whereas the green dots indicate the *Streptococcus* core genome size for each genome comparison. The median values were connected to represent the relationship between number of genomes and gene families. Curves for Sg (**b**) and Ss (**d**) illustrate the number of expected new genes detected with every increase in the number of *Streptococcus* genomes.

Gathering all the functional genes of 14 strains of Sg, we determined a total number of 4,401 pangenomic gene families of Sg. The accessory gene families contributed a larger part of the pan-genome composition (2,774 genes) than the core gene families (1,627 genes). The accessory gene families were further classified into 1,968 dispensable genes (shared by 2 to 13 strains) and 806 strain-specific genes (shared by only one strain). The core gene families of Sg accounted for approximately 37.0% of the total gene families. Due to the low number of Ss isolated strains (5 strains), we included 22 other Ss genomes from the public NCBI database in this analysis in order to have a better representation of this species as a whole. These were all the Ss genomes available at the time of conducting the analysis. Based on the 27 Ss strains, a total of 5,100 pangenomic gene families were identified. The core gene families comprise 1,739 genes (34.1%) and the remainders are accessory gene families. Of the 3,361 accessory gene families, 7% are strain-specific. The pan-genome and core-genome sizes of Ss and Sg were estimated by extrapolation of the above genome data. Briefly, we calculated the gene clusters and core gene families of *Streptococcus* genomes, represented by N (N = 1, 2, 3 ….. 25, 26, 27). All permutations of genome comparisons for every pan-genome size and core genome of N genomes were analyzed to avoid random bias. Simultaneously, their mean values were predicted and depicted along the core genome family curve and pan-genome family curve. The generated pan-genome curves of both Sg and Ss are well-represented by the Heaps law mathematical functions: Y = 573.705131118841 X^0.603^ + 1559.42450454357 and Y = 816.330402837524 X^0.455^ + 1410.909236541, respectively, where Y refers to the pan-genome size while X refers to the number of sequenced *Streptococcus* genomes. According to these equations, the pan-genome size (Y) of both Sg and Ss appeared to reach infinity when the number of genomes (X) increase to infinity (Fig. 2a and c). Therefore, our data suggest that both Sg and Ss have open pan-genomes, which indicates that both species have infinite genomes.

For Sg, the rate of new discovery stabilizes at approximately 110 new genes per additional new genome (Fig. 2b). For example, 295 new genes were detected when a second genome was added to the first Sg genome. The mathematical equation predicted 119 new genes gained by the Sg species with every new Sg genome added. For Ss, we estimated about 61 new genes detected when each additional genome is added (Fig. 2d). Here, we inferred that Sg and Ss have approximately 34–37% of core genes of their total gene clusters, probably inclining to an open pan-genome. The infinite pan-genome of Ss and Sg suggests the bacteria will keep acquiring new genes as they evolve independently over evolutionary time. The intake of new genes may alter the bacterial genome structure and facilitate adaptation of *Streptococcus* species to a dynamic or changing niche^12^.

### Orthologous gene family comparisons

**Figure 3:**
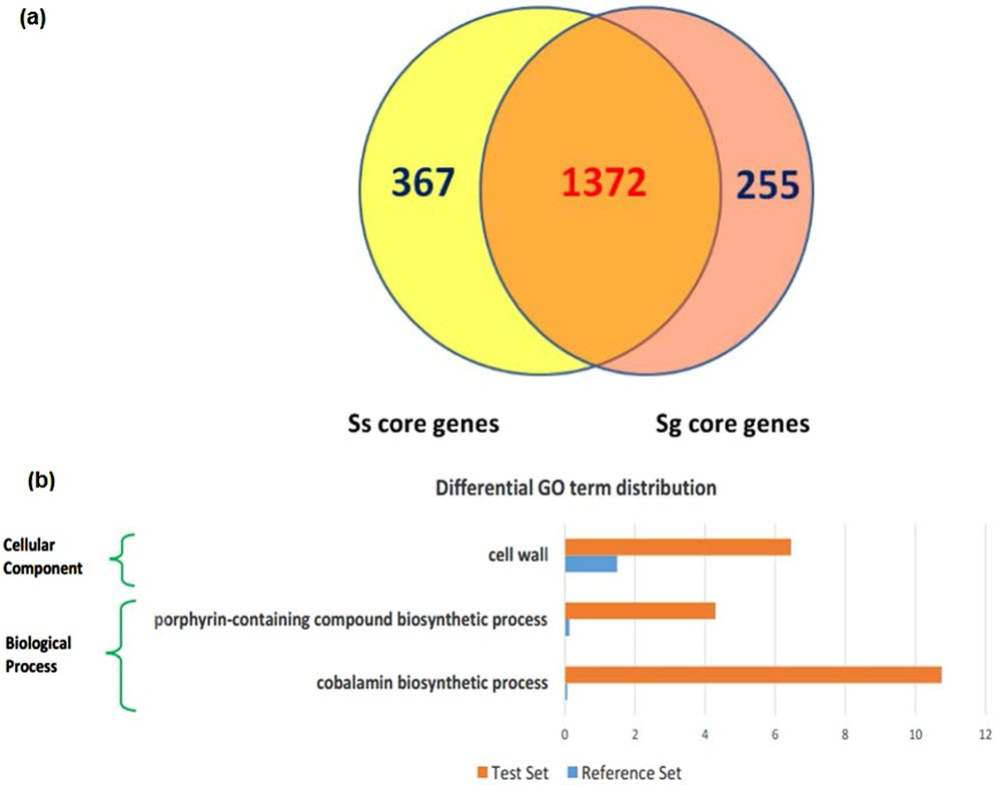
Venn diagram of comparative analysis of orthologous genes in Sg and Ss (**a**) and functional enrichment analysis of unique core genes (**b**). These species share a high number of core genes. Ss has relatively higher species-specific genes compared to Sg. The functional enrichment analysis indicates Ss unique core genes (orange bars) are statistically enriched in two conserved biological processes: cobalamin biosynthesis and biosynthesis of porphyrin-containing compounds. Ss SK36 genes were used as background dataset for comparison.

To identify the overlap between the predicted gene functions within the Sg and Ss genomes, we clustered all predicted genes from both species that were generated during the pan-genome analysis. We compared the core genes of Sg and Ss and found they shared a large set of gene families (1,372), reflecting a high similarity between the two species. Notably, Ss has a relatively higher number of unique core gene families (367) compared to unique core genes of Sg (255) (Fig. 3a).

To examine the biological functions of unique core genes, we performed a functional enrichment analysis using Blast2GO software^13^. We found no statistically enriched functions of unique core genes of Sg. In contrast, we found the unique core genes of Ss are significantly over-represented in porphyrin-containing compound biosynthetic processes and the cobalamin biosynthetic process (Fig. 3b).

The porphyrin-containing compound biosynthetic pathway leads to biosynthesis of porphyrin-containing compounds such as heme or siroheme^14^. In Ss (NCTC 7863), the superpathway of heme biosynthesis includes a number of branch points that lead to biosynthesis of a variety of important compounds such as vitamin B_12_ (cobalamin), siroheme and heme D^15^. Eight genes involved in the porphyrin-containing compound biosynthetic pathway were identified in the unique core genome of *S*. *sanguinis* (Table S1). Four of these genes encode enzymes predicted to be involved in the biosynthesis of uroporphyrinogen III from glutamyl-tRNA: glutamyl-tRNA reductase (EC 1.2.1.70), glutamate-1-semialdehyde aminotransferase (EC 5.4.3.8), porphobilinogen deaminase (EC 2.5.1.61) and uroporphyrinogen III synthase (EC 4.2.1.75). Therefore, the ability to synthesise uroporphyrinogen III appears to be conserved among Ss strains.

Uroporphyrinogen III is the first macrocyclic intermediate in the biosynthesis of tetrapyrroles. In *S*. *sanguinis* it is likely that uroporphyrinogen III is particularly important for cobalamin biosynthesis since genes encoding all components of the cobalamin biosynthetic pathway were present in the unique core genes of Ss. Interestingly, two types of gene clusters, *cobCMTU* and *cbiACDGHKMNP* are primary cobalamin (vitamin B12) biosynthesis genes which have been well-characterized in *Salmonella* Typhimurium^16^. The *cbi* genes located at the 5′ end of the operon are devoted to synthesis of the corrin ring while the *cob* genes located at the 3′ end of the operon are required for the assembly of the nucleotide loop of cobalamin^17^. Cobalamin is required as a cofactor in the enzymatic pathways for degradation of ethanolamine into ammonia and acetaldehye and breakdown of propanediol. Previous studies have reported that cobalamin can enable different bacterial species to obtain carbon and nitrogen in anaerobic conditions within the host when ethanolamine and propanediol are abundant^18^.

Cobalamin is a cobalt-containing vitamin and genes associated with cobalt/nickel uptake *cbi/nikMNQO* were also present in the unique core genome of Ss. These were functionally annotated under the membrane transport group. This gene cluster was first identified in the genome sequence of Ss SK36^19^. These genes are encoded within the upstream region of the cobalamin biosynthesis genes in bacterial genomes including Ss^20^. Previous research reported that the periplasmic binding protein NikA and ATPase NikE transporters from the NikABCDE system of *Escherichia coli* belong to the nickel/peptide/opine ABC transporter family^21^. The *cbiMNQO* operon encodes an Energy Coupling Factor (ECF) transporter. These systems are a subgroup of ABC transporters and CbiMNQO is essential for cobalt and nickel uptake in bacteria^22^. Moreover, the transport of nickel and cobalt along with cobalamin synthesis is particularly important in bacteria to support survival in host environments^23^. Hence, cobalamin synthesis and high-affinity cobalt/nickel uptake might contribute to the survival and growth of *S*. *sanguinis* in dental plaque and/or to its ability to cause infective endocarditis^19^.

### Comparative prophage analysis

**Figure 4:**
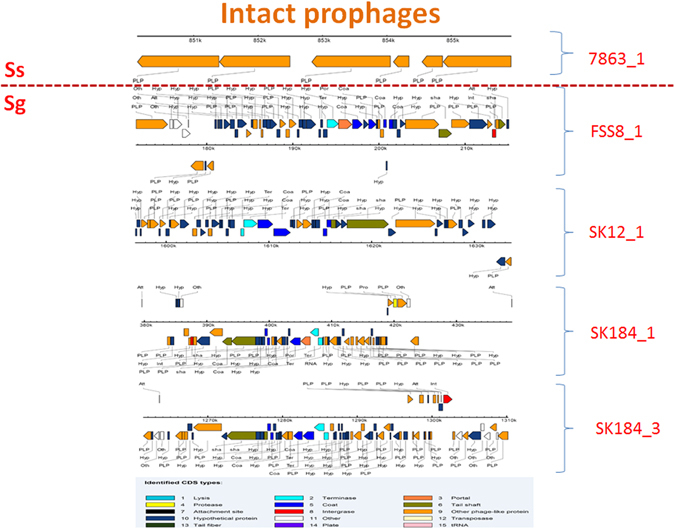
Intact prophages in Sg and Ss. 5 intact prophages were detected, of which four were present in Sg (FSS8_1, SK12_1, SK184_1 and SK184_3) and only one was found in Ss (7863_1).

Prophages may carry new genes that play important roles in the acquisition of new traits and the generation of genetic diversity^24^. Prophages in the genomes of Sg and Ss were predicted using the Phage Search Tool (PHAST) software^25^. In total, twelve putative prophages were identified: eight in Sg and four in Ss. These included five intact prophages, four of which were Sg strain-specific and one was Ss strain-specific (Fig. 4). Only two prophages (FSS4_1 and MB451_1) are conserved across all Ss strains. In addition to phage protein orthologs, two attachment sites: *attL* and *attR* and ancillary enzymes such as integrase were detected in most of these prophages, providing further evidence that they were acquired by horizontal gene transfer (Table S2).

Interestingly, an operon composed of the *efeUOB* system along with genes of the twin-arginine translocation (Tat) pathway, *tatA* (Sec-independent protein secretion pathway component) and *tatC* (Sec-independent protein translocase) was found within the conserved prophage FSS4_1 in all 6 Ss genomes including the reference genome of Ss SK36. The EfeUOB system can import ferrous iron under acid conditions whereas the Tat system exports folded proteins across bacterial cytoplasmic membranes^26, 27^. *Streptococcus thermophilus* was the first *Streptococcus* species reported to possess genes of the Tat system. Subsequently, *tatA* and *tatC* genes were detected in Ss SK36, encoded by SSA_1133 and SSA_1132, respectively^19, 26^. Therefore, we suggest that the acquisition of the FSS4_1 prophage containing the *efeUOB-tat* operon by Ss occurred early after the separation of Ss from Sg.

Another conserved prophage MB451_1 found in Ss contains a gene encoding N-acetylmuramoyl-L-alanine amidase, a streptococcal phage lysin found in Streptococcal C1 bacteriophage^28^. This enzyme hydrolyzes the N-acetylmuramoyl-l-alanine amide bond between the glycan strand and the cross-linking peptide of peptidoglycan^29^. We then utilized the Phage Classification Tool Set (PHACTS), which is an online computational tool, to classify the lifestyle of the MB451_1 prophage^30^. PHACTS predicted the prophage MB451_1 to have a temperate lifestyle (including both lytic and lysogenic phases) with an averaged probability of 0.55 and standard deviation of 0.045. Hence, we deduced that the lysogenic phase enables prophage MB451_1 which carries N-acetylmuramoyl-L-alanine amidase to survive without killing the host.

### Comparative Pathogenomics Analysis

The genetic basis that underlies the transition of oral streptococci from commensals in the mouth to pathogens in infective endocarditis is currently unclear. To identify potential virulence factors of Sg and Ss, we performed a comparative virulence gene profiling analysis using 27 genomes of Ss and 15 genomes of Sg.

We screened for putative virulence genes in all genomes by BLAST searching all protein-coding genes against the Virulence Factor Database (VFDB) with stringent criteria (see Methods). In total, 150 non-redundant virulence genes were identified across all 42 *Streptococcus* genomes. Of the 150 genes, Sg strains possessed 97 to 126 of the virulence genes, whereas Ss strains had 101–139 of the virulence genes (Figure S1). In total, 79 of these genes were shared between Sg and Ss. The common virulence genes include a variety of loci involved in polysaccharide biosynthesis, including homologues of *cps*, *rml* and *rgp* gene clusters. Interestingly, the core loci for polysaccharide production appear to fall into two distinct groups that are fairly evenly distributed across Sg and Ss. This provides further evidence that these species are continually evolving and exchanging genetic material in order to adapt and thrive within the host.

In *Streptococcus pneumoniae*, synthesis of capsular polysaccharides is dependent upon a large gene cluster that consists of four regulatory genes followed by serotype-specific *cps* genes^31^. This locus encodes the machinery required to synthesize and export capsular polysaccharides from the cell. Oral streptococci generally do not produce clear capsules *in vitro*, but most strains examined to date include homologous loci with four regulatory genes upstream of genes for polysaccharide biosynthesis and export. In many oral streptococci, including strains of Sg and Ss, these genetic loci mediate production of receptor polysaccharides (RPSs) that participate in cell-cell adhesion (coaggregation) with other oral bacteria^32^. The structure and function of these RPSs are determined by the precise combinations of transferases and polymerases present in a particular strain. For example, Sg 38 and Ss SK45 contain similar *rps* gene clusters located downstream of the *nrdG* gene but produce antigenically distinct RPSs, probably due to the presence of glycosyl transferases encoded by *wefB* and *wefC* in Sg38, compared with *wefH* in Ss SK45^32, 33^. Polysaccharides produced by some strains of Sg and Ss, including Sg Challis and Ss SK36, are not involved in coaggregation. Disruption of the polysaccharide gene locus in Sg Challis abrogated adhesion to collagen type I or II, indicating that the Sg Challis polysaccharide may be more important for the recognition of host tissue rather than other bacteria^34^.

**Figure 5:**
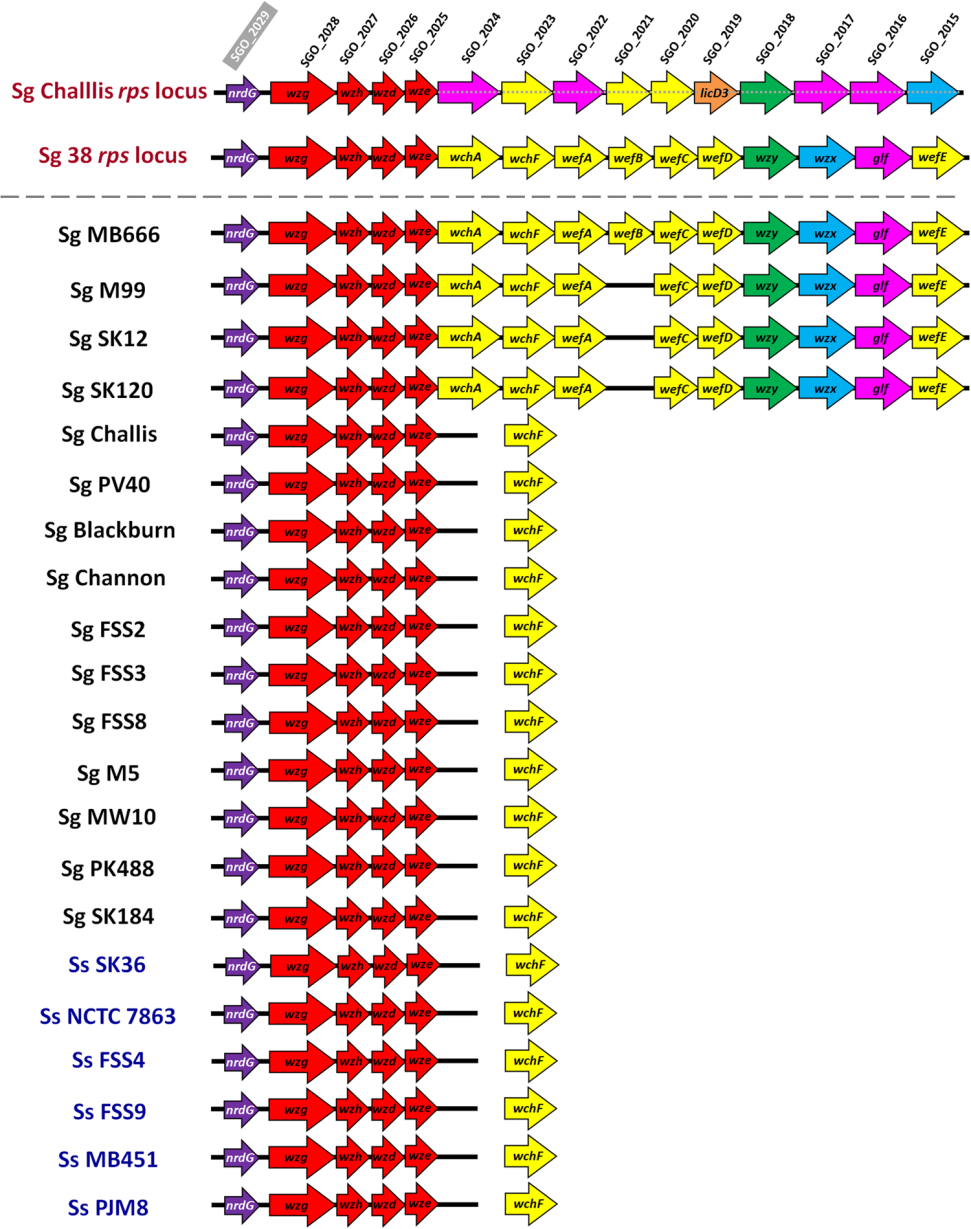
Illustration of rps/polysaccharide gene clusters of Sg 38 and Sg Challis in *Streptococcus* genomes. Color coding is as follows: *nrdG* gene upstream of the polysaccharide gene cluster (purple), regulatory genes (red), transferases (yellow), putative phosphorylcholine transferase *licD3* (orange), polysaccharide polymerases (green), flippases (blue), nucleotide-linked sugar synthesis (magenta).

Closer examination of genome sequences in the strains presented here identified *rps* gene clusters similar to those of Sg 38 and Ss SK45 in several Sg strains but not in Ss (Fig. 5). Only Sg MB666 contained *wefB*, whereas Sg M99, SK12 and SK120 contained similar gene clusters without *wefB*. All other streptococci sequenced here contained the first four genes downstream of *nrdG* (*wzg*, *wzh*, *wzd* and *wze*) but lacked clear homologues of the Sg 38 genes *wchA*, *wchF*, *wefA*, *wefB*, *wefC*, *wefD*, *wzy*, *wzx*, *glf* and *wefE*. Homologues of *wchF* were identified, but these were always at a separate locus from *nrdG*-*wze*. Analysis of the Sg Challis genome region downstream of *wze* identified a number of other putative glycosyltransferases and polysaccharide production enzymes that have not yet been well characterized (Fig. 5).

The Mauve genome analysis tool separated the Sg Challis polysaccharide biosynthesis locus into 9 locally contiguous blocks (LCB’s) (Figure S2). All of these were present in the same order in Sg strains PV40, FSS3, Blackburn, MW10, SK184, PK488 and FSS2. Sg FSS8 lacked a large central region containing 5 LCB’s. Sg Channon displayed an absence of a smaller region of 2 LCB’s and Sg M5 was missing a region of 2 LCB’s at the 3′ end of the locus. All Ss strains shared the core polysaccharide locus structure with Sg Challis, with the exception that they lacked the 3′ LCB. Moreover, the Sg 38-type *rps* loci in Sg MB666, SK12, SK120 and M99 were clearly distinguishable from Sg Challis in the Mauve analysis (Figure S2).

Since the Sg Challis-type polysaccharide gene cluster structure is so widely conserved, we propose that this is the ancestral gene cluster in Sg and Ss strains. Presumably, the Sg 38-type gene cluster arrangement has arisen at least twice by horizontal gene transfer since it is present in at least one strain of both Sg and Ss, although it was not observed in any Ss strains analyzed here. It is notable that the strains harboring Sg 38-type *rps* gene loci did not cluster together by either 16S rRNA or whole genome SNP analysis (Fig. 1). Nevertheless, this does not exclude the possibility that these strains have diverged from a common ancestor after acquiring the Sg 38-type *rps* locus.

Genes encoding enzymes involved in the production of key substrates for polysaccharide biosynthesis are located at a number of loci that are distinct from the polysaccharide biosynthesis/export operons. For example, dTDP-L-rhamnose, is synthesized by the products of the *rml* genes. Of these, *rmlACB* are located downstream of *gufA* whilst *rmlD* is on a separate operon downstream of *orf15* ^32^. These *rml* genes appear to be conserved in Sg and Ss strains, indicating that they play key functions in the metabolism of these species. The *rml* genes, together with *rgp* genes, may also be involved in the synthesis of other rhamnose glucose polymers (RGPs) that have been identified in a range of streptococci^35^. In *Streptococcus suis*, RGPs have been linked to several pathology-induced functions such as triggering sepsis, stimulating release of inflammatory cytokines and provoking nitric oxide production^36^. RGPs of oral streptococci have been shown to stimulate platelet aggregation, a process that is thought to be important in the pathogenesis of streptococcal infective endocarditis^37^. The RGPs also play significant roles in assisting bacteria to escape killing by human polymorphonuclear leukocytes^38^. Overall, the synthesis of RGPs by Ss and Sg may contribute to their pathogenesis in infective endocarditis, as well as modulating initial adhesion during the colonization of tooth surfaces and the formation of dental plaque.

The ability of Sg to adhere to host surfaces and tissues is thought to be important for colonization of the oral cavity, as well as attachment to endothelial tissue and platelet binding in infective endocarditis. A family of serine-rich repeat glycoproteins plays a key role in adhesion to glycosylated host substrates including platelets^39^. These polypeptides have an N-terminal binding region (BR), a long highly repetitive serine-rich domain and a C-terminal LPxTG cell wall anchor. Variants of BRs have been described that have distinct specificities for host substrates. The SrpA-type is found in Ss strains, whereas Hsa and GspB variants are each present in different subsets of Sg strains. Several of the Sg strains employed in the current study have been assessed for their ability to induce platelet adhesion^40^. In general, the level of Hsa expressed on the cell surface correlated with platelet binding levels, whereas the association was not so clear for strains that produce the GspB variant. Using PCR primers specific for the *hsa* or *gspB* BR-encoding sequence we identified one strain, Sg PK488, which did not appear to have either variant (data not shown). Nevertheless, Sg PK488 was shown to bind the model sialoglycoprotein fetuin in a sialic acid-dependent manner (Figure S3). Therefore, to gain a better understanding of the distribution of serine-rich repeat proteins in Sg and Ss, we drew a phylogenetic tree based on the BRs of serine-rich repeat proteins predicted from the whole genome sequences (Figure S3). All but one of the Ss strains had an SrpA-type BR, whereas the majority of Sg strains clustered with the Hsa variant or the GspB type. However, the BRs of Sg PK488 and Ss FSS4 did not fall within any of these clusters. They appeared to be more closely related to GspB than to Hsa or SrpA. It is not clear whether the unusual BRs of Sg PK488 and Ss FSS4 have arisen through horizontal gene transfer. We noted that the region upstream of the genes encoding serine-rich repeat proteins was different in Sg compared with Ss. In all Sg strains, this region contains genes involved in pyridoxine metabolism, including a predicted regulator, *pdxK* encoding pyridoxal kinase and *pdxU* encoding a pyridoxine transporter. By contrast, in all Ss strains including FSS4, there is a serine tRNA immediately upstream of the gene encoding the serine-rich repeat protein. Therefore, it appears that a genome rearrangement event likely occurred around the time of the speciation event that separated Sg from Ss. It will be interesting to determine whether they confer a different binding specificity from other BRs that may influence their ability to colonize host tissues or adhere to platelets.

**Figure 6:**
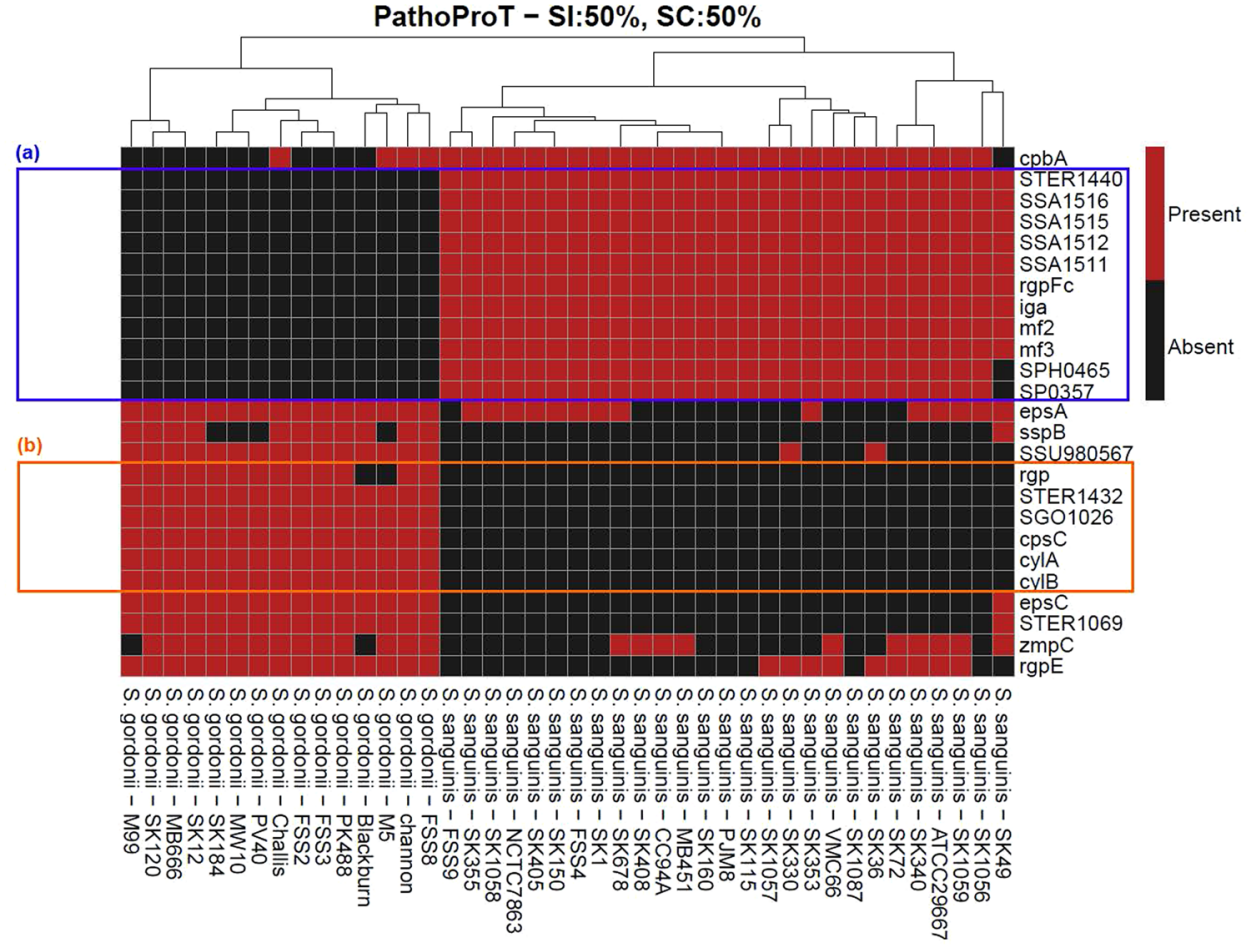
The screenshot of heatmap shows the main differences of virulence genes harbored by Ss and Sg. The blue box (**a**) shows the unique virulence genes of Ss while the orange box (**b**) depicts the unique virulence genes of Sg.

Figure 6 shows the main differences in putative virulence genes between Sg and Ss. Virulence-associated genes present uniquely in Ss include *SSA1511*, *SSA1512*, *SSA1515* and *SSA1516*, which encode hypothetical membrane proteins and glycosyltransferases. Additionally, *mf2* and *mf3* (mitogenic factor 2 and 3), which were only detected in Ss, encode DNases which have been reported in other streptococci to reduce the viscosity of pus via their enzymatic activity, facilitating the colonization of bacteria across tissue surfaces during invasive streptococcal infections^41^. The virulence gene analysis also identified the *iga* gene among the unique genes of Ss. The *iga* gene encodes IgA protease, and previous studies have shown IgA protease activity in Ss but not in Sg^5^. The IgA protease has been shown to enhance adhesion of oral bacteria to saliva-coated hydroxyapatite^42^. The proteolytic activity of IgA proteases decreases the efficiency of secretory antibodies^43^. However, Fab alpha fragments are generated to sustain the antigen-binding function on the bacterial cell surface, promoting Ss adherence to tissues in the oral cavity^43^. The IgA proteases have exquisite specificity for human IgA, and therefore the presence of IgA proteases in Ss suggests an independent evolution of the enzymes in proteolysis during colonization or infection of humans^44^.

Strikingly, the Sg-specific *cyl* gene cluster appears to be unique to Sg and the β-haemolytic Group B streptococci^45^. Together, *cylA* and *cylB* encode an ATP-binding cassette (ABC) transporter^46^ that plays important roles in antibiotic resistance as multidrug resistance (MDR) transporters in addition to its core function as an exporter of the Cyl cytolysin^47^. We investigated the homologs of *cylA* and *cylB* genes and found three homologs for each gene in *Streptococcus agalactiae*, which currently annotated as hypothetical proteins, *cylA/cylB* proteins and *cylA/cylB* permeases separately. We assessed the completeness of the *S*. *agalactiae cyl* genes against the Sg *cyl* genes. There was remarkably high sequence coverage and sequence identity for *cylA* and *cylB* genes which were (100/78.64)% and (100/80.82)%, respectively. To further verify this finding, we also tested the sequence coverage of *cyl* genes in the complete whole-genome of Ss SK36 and the results showed that *cyl* genes are likely to be absent in Ss genomes. Given the presence of these genes in all Sg strains, this may provide the first evidence of CylA and CylB production by the α-haemolytic Sg. The role of CylA/B in multidrug resistance in Sg remains to be determined.

### Comparative Genomic Island (GI) analysis

Oral streptococci encounter significant fluctuations in environmental conditions such as surrounding pH, oxygen tension or osmolarity when growing in dental plaque. The transition to the bloodstream environment involves an even greater shift in the conditions of the external environment. We postulated that the adaptation and evolution of streptococci to cope with different environments within the human body may have been mediated through the acquisition of gene clusters or GIs by horizontal gene transfer. Typically, GIs in bacteria harbor genes encoding important traits such as antibiotic resistance, symbiosis and fitness^48^. Therefore, horizontally transferred GIs in the genomes of Sg and Ss were predicted using the IslandViewer software tool^49^.

In total, 13 putative GIs were identified: two conserved GIs shared by all Sg and Ss strains, 6 Sg-specific GIs and five Ss-specific GIs (Table 2 and Table S3). For example, GI_55 was found to be conserved in Sg and Ss and is composed of a series of putative V-type ATP synthase subunits (C, E, F, G, I and K) and a GCN5-related N-acetyltransferase (GNAT) family acetyltransferase. V-type ATP synthases are exclusively found in low GC, gram-positive bacteria and utilize the free energy released from phosphoenol pyruvate (PEP) or ATP hydrolysis to pump solutes across the membrane against concentration gradients^50^. A recent report has suggested V-type ATPases in *Streptococcus pyogenes* are regulated by a group of small RNAs. Most V-type ATPases pump hydrogen ions from the cytosol, ensuring the survival of *Streptococcus* species by overcoming acid stress during growth or infection^51^. It is possible that these systems help Ss and Sg to survive cycles of acidification within dental plaque. Alternatively, these systems may pump Na^+^ ions rather than H^+^ since it has been shown that the *Enterococcus hirae* V-type ATPase pumps Na+ ions, and promotes survival in high pH^52^. However, the actual function of this system is still unclear and further work is required to determine the substrate specificity and physiological roles of streptococcal V type ATPases. Overall, it is likely that the acquisition of the 5,516 bp GI_55 by Sg and Ss through lateral gene transfer may have enhanced their ability to survive in low-pH environments such as cariogenic dental plaque.

**Table 2:** Summary of predicted GIs in the genomes of Sg and Ss.

Genomic Island	Size (bp)	Sg														Ss				
		PV40	Blackburn	Channon	FSS2	FSS3	FSS8	M5	M99	MB666	MW10	PK488	SK12	SK120	SK184	NCTC 7863	MB451	PJM8	FSS4	FSS9
GI_5	**5253**	\	\	\	\	\	\	\	\	\	\	\	\	\	\					
GI_14	**10312**	\	\	\	\	\	\	\	\	\	\	\	\	\	\					
GI_16	**5085**	#	#	#	#	#	#	#	#	#	#	#	#	#	#	#	#	#	#	#
GI_31	**5557**															*	*	*	*	*
GI_43	**7035**															*	*	*	*	*
GI_45	**5556**	\	\	\	\	\	\	\	\	\	\	\	\	\	\					
GI_47	**7627**															*	*	*	*	*
GI_51	**7355**	\	\	\	\	\	\	\	\	\	\	\	\	\	\					
GI_53	**4194**															*	*	*	*	*
GI_55	**5516**	#	#	#	#	#	#	#	#	#	#	#	#	#	#	#	#	#	#	#
GI_58	**7364**	\	\	\	\	\	\	\	\	\	\	\	\	\	\					
GI_67	**4094**	\	\	\	\	\	\	\	\	\	\	\	\	\	\					
GI_75	**4183**															*	*	*	*	*

Two conserved GIs were shared by Ss and Sg (marked with hashtag), six Sg*-*specific GIs (marked with backslash) and five Ss-specific GIs (marked with asterisks).

Another conserved GI, GI_16, consists of: iojap (Iowa-japonica) protein, a methyltransferase, a hydrolase from the Haloacid Dehalogenase (HAD) superfamily, *yqeK* gene and nicotinate-nucleotide adenylyltransferase. In bacteria, the *ybeB* gene is the ortholog of *iojap* protein which usually forms a conserved operon with the *ybeA* gene encoding a predicted methyltransferase. This *ybe* operon gene is often found adjacent to the *nadD* gene encoding nicotinate-nucleotide adenylyltransferase in nicotinamide-adenine dinucleotide (NAD) biosynthesis^53^. Additionally, this *ybe* operon has been reported to have an overlapping coding region with the *yqeK* gene, encoding a metal-dependent phosphatase^54^. Together, *nadD* and *ybeB* appear to form a two-domain fusion protein^53^. Hence, we deduced the methyltransferase found in GI_16 is a likely a homologue of the *ybeA* gene which shares an operon with *ybeB* gene. However, the significance of the association between *yqeK* and *nadD* as well as the structural terminology of *nadD-YbeB* complex remains unknown.

Out of the six Sg-specific GIs detected, GI_67 is comprised of genes *camG*, encoding a putative lipoprotein, and *parE*, encoding topoisomerase IV subunit B. The *camG* gene encodes a lipoprotein, with a leader sequence that includes a 7-amino acid peptide pheromone known as gordonii-cAM373 heptapeptide SVFILAA^55^. This pheromone is required for transfer of plasmid DNA from *Enterococcus faecalis* into Sg and has been associated with multiple antibiotic resistance^55^. We hypothesize that genes on GI_67 may facilitate the exchange of antibiotic resistance genes between oral bacteria within dental plaque.

Interestingly, the putative Sg*-*specific GI_45, GI_51 and GI_58 which vary in size from 5,556 to 7,364 bp share a large group of paralogous genes. The *com* gene cluster, *comCDE*, is located in all three putative GIs. These genes encode a peptide pheromone (*comC*) and a sensing system (*comDE*) that are involved in quorum sensing, transformation and biofilm formation^56, 57^. Inactivation of *comD* and *comE* leads to abnormal biofilm formation which eventually decreased plaque biomass^57, 58^. Hence, the competence regulation operon found in GI_45, GI_51 and GI_58 of Sg activates streptococcal cell-cell peptide signaling systems of Sg via exogenous DNA incorporation, enabling acid tolerance of Sg in oral biofilm formation^59^. Apart from its role in oral biofilm formation, *comCDE* has also been implicated in increasing genome plasticity via uptake of new genes^60^, DNA repair^61^, as well as providing nutrition of carbon, nitrogen, phosphorus, and energy source for Sg^62^. It is likely that the presence of multiple *comCDE* systems may enhance the capacity of Sg to uptake genetic material, and increase its rate of evolution. Within GI_45, GI_51 and GI_58 we identified another streptococcal plasmid acquired gene, *parB*, which is associated with important biological processes of DNA replication, cell division and cell growth^63^. In other bacteria such as *Vibrio cholerae* and *Escherichia coli*, *parB* is part of an operon along with the *parA* gene that together have been implicated in drug resistance, stress response, and pathogenesis^64^. It is unclear whether *parB* is important in Sg since *parA* is absent.

Another important gene, present within GI_45, GI_51 and GI_58, is the *degP/htrA* gene, which encodes a protein responsible for folding, maturation and degradation of secreted proteins^65^. Recently, the *htrA* gene has been shown to play a key role in the repair of reactive oxygen species (ROS)-damaged DNA and protein^66^. The accumulation of misfolded proteins causes the susceptibility of bacteria towards high temperatures and reactive oxygen intermediates stresses. In *S*. *pyogenes*, *degP* gene knockout is impaired in virulence in a mouse model of streptococcal infection^67^. Therefore, the presence of *degP/htrA* may enable Sg to overcome thermal, oxidative and osmotic stresses, thus indirectly enhancing its virulence in infections.

We identified five putative Ss*-*specific GIs known as GI_31, GI_43, GI_47, GI_53, and GI_75. Of these, GI_31 is a particular concern since it carries a permease of the drug/metabolite transporter (DMT) superfamily and a TetR/AcrR family transcriptional regulator (TFR), and thus is potentially an antibiotic resistance island. The DMT Superfamily which consists of 35 distinctive subfamilies is associated with multi-drug and various antibiotic resistances^68^. In addition, the TFRs have been reported to be overarching regulators involved in numerous processes including biosynthesis or degradation of fatty acids^69^, antibiotic biosynthesis or activation^70^, biofilm formation^71^, toxin production^72^, and cell-cell signaling^73^.

We also found an intrinsic putative GI_47, which houses different functional gene components, within six genomes of Ss. This GI includes a GNAT acetyltransferase that may convey aminoglycoside resistance. A ribosomal RNA small subunit methyltransferase E (*rsmE*) is also found in GI_47. This gene encodes an enzyme that methylates DNA, RNA, proteins or small molecules such as catechol and is also associated with antibiotic resistance^74, 75^. In addition, GI_47 includes the “housecleaning” gene *mutt* encoding a nudix family protein that catalyzes pyrophosphohydrolase activity directed at the removal of mutagens arising from inappropriate methylation by *rsmE* as well as reactive oxygen species (ROS) generated by endogenous metabolites^76^. Two mobile elements and an integrase found within this putative GI_47 provide evidence that this region has been horizontally transferred to Ss.

Two putative Ss*-*specific GIs, GI_53 and GI_75, were found to include genes encoding CAAX amino protease family members and TetR family transcriptional regulators (TFR). Two genes, *bfrH1* and *bfrH2* encode CAAX family proteins. In Ss, these two genes are regulated by the BfrABss two-component system which controls the expression of two *bfrCD*-homologous operons (*bfrCDss* and *bfrXYss*), a *bfrH*-homologous gene (*bfrH1ss*) and another CAAX amino-terminal protease family protein gene (*bfrH2ss*). Homologues of this BfrABss system are required for biofilm formation by oral streptococci^77^. According to a recent report from Jimin and colleagues^78^, Ss has the highest known level of CAAX amino protease compared to other species. It is likely that these CAAX effector proteases are important for the biological function of Ss, perhaps by contributing to establishment and survival within dental plaque.

### Antibiotic Resistance analysis

Based on the genomic island (GI) analysis, we found that many of the genes on the GIs have been associated with antimicrobial resistance, including GNAT acetyltransferases, *parE*, and TetR family regulators. For example, GNAT acetyltransferases have been associated with reistance to aminoglycosides such as gentamicin^79^. Variants of *parE*, along with *gyrA*, *gyrB* and *parC*, are associated with elevated resistance to fluoroquinolones such as ciprofloxacin^80^. TetR-family regulators are often responsible for up-regulation of multi-drug effleux pumps, leading to resistance to many different antibiotics^81^. Therefore, we tested the resistance of all the newly sequenced Sg and Ss strains to nine different types of antibiotic: erythromycin, trimepthoprim, sulphamethoxazole, tetracycline, penicillin G, clindamycin, gentamicin, fusidic acid and ciprofloxacin. All Sg and Ss strains were sensitive to erythromycin, penicillin G, clindamycin and ciprofloxacin, moderately sensitive to gentamicin and fusidic acid and resistant to trimethoprim and sulphamethaxazole. Interestingly, five strains (Ss MB451 and Sg strains PV40, FSS2, MB666 and MW10) were found to be resistant to tetracycline, whereas all other strains were sensitive. Genome analysis identified the *tetM* gene in all the tetracycline-resistant streptococcal strains and not in any of the other strains, indicating the acquisition of the *tetM* resistance determinant likely has conferred tetracycline resistance in *S*. *sanguinis* and *S*. *gordonii* strains. Nevertheless, there was no evidence that genes present on genomic islands in Sg or Ss were responsible for harboring antibiotic resistance determinants.

In conclusion, our comparative genome analyses provide insights into the differing ecological strategies of Sg and Ss. Both species are common within dental plaque and both have the potential to cause infective endocarditis. However, Ss is usually present in higher numbers than Sg, and differing associations between these species and oral disease have been shown. Functions such as cobalamin biosynthesis, IgA protease activity and CAAX proteases may contribute to the expansion of Ss within dental plaque. On the other hand, the presence of *cylA* and *cylB* within the core genome of Sg is interesting and warrants further study. There are no genes that are clearly enriched in endocarditis isolates, and this is in keeping with the observation that oral and endocarditis isolates of Ss do not form distinct subclones^82^. It is clear that both Sg and Ss have open pan genomes and these species continue to evolve and acquire new genes. Potentially, the exchange of genetic information between bacteria in biofilms may accelerate the spread of antibiotic resistance between bacteria in the oral cavity. Overall, our comparative analyses of Sg and Ss will provide a basis for understanding how these species establish within dental plaque and how they transition from commensal species within the mouth to important pathogens in infective endocarditis.

## Methods

### Bacterial isolation and DNA extraction

The 19 strains of Ss and Sg included in this study (PV40, NCTC7863, Blackburn, Channon, FSS2, FSS3, FSS4, FSS8, FSS9, M5, M99, MB451, MB666, MW10, PJM8, PK488, SK12, SK120 and SK184) were originally isolated from four different geographical regions. Of these, thirteen were originally isolated from the United Kingdom, four from the United States and one each from Denmark and Australia. Six strains originated from oral cavity samples; ten strains were from subacute bacterial endocarditis and the origin of the other three is not known. All *Streptococcus* strains were cultured in THYE medium (30 g/L Todd Hewitt broth, 5 g/L yeast extract) for 16 hours at 37 °C prior to DNA extraction.

### Library preparation and next-generation sequencing

Chromosomal DNA was extracted as previously described^83^. Libraries were prepared by fragmentation of DNA samples using a Covaris S2 ultrasonicator for 120 sec at 5.5–6.0 °C. The quantity and quality of the fragmented DNA were evaluated using an Agilent BioAnalyzer 2100. The sample was size selected using Invitrogen 2% agarose E-gels. For DNA library construction, only the fragments tagged with adapter molecules at both ends underwent 10 cycles of PCR. The constructed genomic library was validated using an Agilent BioAnalyzer 2100. The 19 *Streptococcus* genomes were sequenced on the Illumina Hiseq 2000 sequencing platform. The paired-end sequencing of *Streptococcus* genomes uses a standard read length of 100 base pairs. The *Streptococcus* genomes were run on a single lane, employing the TruSeq LT assay. The paired-end sequencing generates two FASTQ output data files: one containing the forward primer (“AGATCGGAAGAGCACACGTCTGAACTCCAGTCA”) derived reads “_R1” and one containing the reverse primer (“AGATCGGAAGAGCGTCGTGTAGGGAAAGAGTGT”) derived reads “_R2”. The detailed sequencing results are shown in Tables S4 and S5.

### Raw read quality checking and preprocessing

The raw read quality was verified through FastQC software^84^. The overall genome showed satisfactory results of per base N content and optimal per base sequence quality with no overrepresented sequences. The quality score is directly proportional to the level of base call. Data pre-processing was completed by a trimming approach using CLC Genomic Workbench V6.5 (CLC BIO Inc., Aarhus, Denmark). A series of trimming operations offered by CLC Genomic Workbench V6.5 were employed: quality trimming based on quality scores, ambiguity trimming of gaps in scaffold genomes, adapter trimming, base trimming by removing a specified number of bases at either 3′ or 5′ end of the reads and length trimming within a specified threshold. We selected the quality trimming which applies the modified-Mott trimming algorithm. All genome sequences were trimmed based on Phred quality score Q20 (1/100 bases). The default parameter for quality trimming was applied, allowing a maximum of 2 ambiguities. The trimming approach is crucial in order to ensure adequate stringency of the *Streptococcus* genomic sequences.

### Genome assembly and annotation

The *de novo* assembly was performed using CLC Workbench 6.5 with Phred quality score Q20 (1/100 bases). In general, genome assembly involved the generation of simple contig sequences using the information within the read sequences. The N50 contig was estimated by summarizing the lengths of the largest contigs until half of the total contig length. High N50 values of the genomes and the low contig numbers of genomes are indicative of good genome assemblies. After assembly, the assembled genome sequences were searched against common contaminant databases for contamination screening and any contaminated sequences were removed. To gain better insights into the assembled genomes and to evaluate the completeness of these *Streptococcus* genomes, we mapped all assemblies onto the complete reference genomes of Ss SK36 and Sg Challis using the NUCmer program^85^. Genome annotation of these 19 *Streptococcus* genome sequences was then performed via the fully automated Rapid Annotation using Subsystem Technology (RAST) pipeline^86^. Genes and functional proteins were assigned based on their phylogeny relationship relatedness in FIGfams database subsystem and metabolic pathways.

### Multiple sequence alignment (MSA) and phylogenetic inference

For single gene marker 16S rRNA phylogeny tree, we extracted the predicted 16S rRNA sequences from each *Streptococcus* genome using RNAmmer 1.2 Server^87^. Next, we conducted multiple sequence alignment (MSA) of single gene 16S rRNA sequences using MAFFT web-based program^88^. Core-genome SNP sequences of each *Streptococcus* genome were determined via the Panseq online web-tool^89^. Panseq aligned all genome sequences and identified core/conserved genome regions. SNPs were called within the core genome sequences. Alignments of these core genome SNPs were performed using ClustalW from European Bioinformatics Institute. Ultimately, the generated MSA results from both MAFFT and Panseq servers were then run on MEGA6 (Molecular Evolutionary Genetics Analysis 6) software^90^ in order to build the phylogenetic tree. The phylogeny trees of both 16S rRNA and core-genome SNPs were constructed using 1,000 bootstrapping replications via the Neighbour-Joining (NJ) algorithm method.

### Orthologous gene family comparisons and pan-genome analysis

The pan-genome analysis describes a complete gene set of all strains of a species including the core genome (genes present in all strains), and the accessory genome which comprises the dispensable genome (genes present in two or more strains) and the unique genome (genes specific to single strains). The pan-genome study of the *Streptococcus* isolates was performed using the Pan-Genomes Analysis Pipeline (PGAP) which implements functional ortholog clustering using the amino acid sequences of Ss and Sg based on Gene Family (GF) method^91^. Each amino acid sequence was labelled with specific strain identifiers which are later concatenated into a single input sequence file. Using the BLASTALL algorithm, the minimum score value was set to 50 and E-value to 10^−8^ ^92^. Based on the Markov Cluster Algorithm (MCL), the amino acid sequence cutoff was adjusted to 50% identity and 50% coverage in order to group two genes into the same cluster^93^. Finally, in-house Perl scripts were used to retrieve amino acid sequences of accessory genes and searched against oral *Streptococcus* genomes using TBLASTN. This method was used to identify gene content which could be overlooked by the RAST pipeline.

### Functional enrichment analysis

In order to associate putative biological functions with the unique core genes of Ss and Sg, and to discover unexpected shared functions between these unique core genes, we performed functional enrichment analysis using Blast2GO software^94^. The Blast2GO functional annotation involves three steps: BLAST to find homologous sequences, MAPPING to retrieve Gene Ontology (GO) terms and ANNOTATION to select reliable functions. BLAST was implemented using the amino acid sequences of reference strains Ss SK36 and Sg Challis. After MAPPING and ANNOTATION, we then ran InterPro Scan prior to functional enrichment process. Target lists of Ss and Sg unique core genes were selected respectively for specialized functional enrichment analysis, generating GO graphs from tables of under- and over-enriched *Streptococcus* unique core genes.

### Virulence gene prediction

Virulence genes of Ss and Sg were identified by BLAST searching 42 amino acid sequences of the *Streptococcus* genomes against the virulence factor database (VFDB)^95^. In-house Perl scripts were then used to process BLAST outputs (generated by searching these query sequences against VFDB) for each RAST-predicted protein (query sequence) in the oral *Streptococcus* genomes. The filtered BLAST results were consolidated and virulence genes with minimum mapped sequence identity and sequence coverage of 50% in both query and subject were organised in a matrix table. Lastly, in-house R scripts were used for hierarchical clustering and a heat map was generated for visualization. Predicted virulence genes are highlighted in red in heat map (Figure S1), indicating the presence of virulence genes in *Streptococcus* species.

The *rps* locus genes prediction on 19 strains of Sg and Ss was performed manually using our in-house scripts. The protein sequences of the first four regulatory genes: *wzg* (gi|157075510|gb|ABV10193.1|:1–486), *wzh* (gi|157075683|gb|ABV10366.1|:1–243), *wzd* (gi|157076133|gb|ABV10816.1|:1–231) and *wze* (gi|157076456|gb|ABV11139.1|:1–231) were extracted from the Sg Challis genome stored in the National Center for Biotechnology Information (NCBI) resource, while the amino acid sequences of the 10 genes: *wchA* (Q83YQ3), *wchF* (Q83YS0), *wefA* (Q83YR9), *wefB* (Q83YQ5), *wefC* (Q83YR8), *wefD* (Q83YR4), *wzy* (Q83YR3), *wzx* (Q83YR2), *glf* (A0A0F2CL65) and *wefE* (Q83YR0) were retrieved from the same species genome available on UniProt resource. Next, we performed protein BLAST using these *rps* locus gene sequences against *Streptococcus* protein sequences. The protein BLAST results were then filtered based on the threshold of 50% sequence identity and 50% sequence coverage. To determine whether similar genome arrangements were present in other Sg and Ss strains, genomes were analyzed for the presence of ‘locally collinear blocks’ (LCBs) via the Mauve genome analysis tool^96^.

### Comparative prophage analysis

The 12 different putative prophages of Sg and Ss were identified using PHAST (Phage Search Tool) web server^25^. The assembled contig sequences of the *Streptococcus* species were concatenated to serve as input files for the prophage prediction by the PHAST. The identification and completeness of these putative prophages were evaluated through a series of operations including genome-scale ORF prediction and translation via GLIMMER, protein, phage sequence and tRNA identification, attachment site recognition and gene clustering density measurements as well as sequence annotation text mining. The predicted putative prophages were eliminated if the prophages were located within two different contigs. All putative prophages were then BLAST searched across strains of Ss and Sg for genome completeness checking to verify their presence in oral streptococcal genomes with nucleotide sequence identity cutoff values of 70% identity and 70% coverage. An intact prophage was defined by achieving scores ≥90 by PHAST. To predict the lifestyle of the prophage, we utilized the Phage Classification Tool Set (PHACTS)^30^ which involved a novel similarity algorithm and Random Forest Classifier. The file which contains the protein sequences of the predicted genes in the phage as uploaded for phage lifestyle annotation using a similarity algorithm. Datasets consisting of various sizes of partial proteomes were created. Each proteome was created by randomly selecting a replacement phage with a known lifestyle followed by randomly choosing a set of contiguous proteins in that phage. Lastly, classification of the lifestyle of a phage (‘virulent’ or ‘temperate’) is performed by Random Forest classifier.

### Comparative Genomic Island (GI) analysis

The putative GIs in Ss and Sg were predicted by the IslandViewer software tool^49^ which involved three different GI identification approaches: sequence composition-based approaches using SIGI-HMM and IslandPath-DIMOB, and the comparative genomics approach using IslandPick. The predicted GIs were then further filtered by removing GIs with genomic length less than 10 kbp. Likewise, the predicted putative GIs from IslandViewer were further inspected by omitting GIs that mapped across two different contigs. We utilized BLASTClust to cluster similar GI sequences, with parameters set so that any two GIs with at least 50% sequence identity and 50% sequence coverage would be clustered together.

### Antibiotic resistance testing

Resistance to penicillin G, clindamycin, gentamicin, fusidic acid, erythromycin, trimethoprim, sulphamethoxazole and tetracycline was tested using an M43 Mastring (Mast Group Ltd, Bootle, UK) in accordance with the manufacturer’s instructions. Briefly, bacteria were cultured for 16 h in THYE broth, and suspensions of cells (100 µl) were spread over the surface of solidified THYE medium. A Mastring was placed on the plate, and incubated for 48 h at 37 °C. The zone of diffusion was measured. Strains were considered resistant if zones of clearance were <1 mm, intermediate where zones were 1–5 mm and sensitive if zones were >5 mm. A similar disk diffusion test was used to assess resistance to ciprofloxacin, using individual disks impregnated with 0.002 µg/ml ciprofloxacin (MA0104, Thermo Fisher).

## Electronic supplementary material

Supplementary Information
