# Supplementary material for: Distinct Biological Potential of Streptococcus gordonii and Streptococcus sanguinis Revealed by Comparative Genome Analysis
Source: Sci Rep. 2017 Jun 7;7:2949. doi: 10.1038/s41598-017-02399-4 (PMC5462765; doi:10.1038/s41598-017-02399-4)
Supplement: Supplementary file 1 — Supplementary Information [file 41598_2017_2399_MOESM1_ESM.pdf]

Distinct Biological Potential of *Streptococcus gordonii* and *Streptococcus sanguinis* Revealed by Comparative Genome Analysis

Wenning Zheng[1,2]**; Mui Fern Tan**[1,2]**; Lesley A. Old**[4]**; Ian C. Paterson**[2,3]**; Nicholas S Jakubovics**[4,5*]**; Siew Woh Choo**[1,2*]

[1]Genome Informatics Research Laboratory, High Impact Research Building (HIR) Building, University of Malaya, 50603 Kuala Lumpur, Malaysia

[2]Department of Oral and Craniofacial Sciences, Faculty of Dentistry, University of Malaya, 50603 Kuala Lumpur, Malaysia

[3]Oral Cancer Research and Coordinating Centre, Faculty of Dentistry, University of Malaya, 50603 Kuala Lumpur, Malaysia

[4]Centre for Oral Health Research, School of Dental Sciences, Newcastle University, Newcastle upon Tyne, NE2 4BW, United Kingdom

[5]Genome Solutions Sdn Bhd, Suite 8, Innovation Incubator UM, Level 5, Research Management & Innovation Complex, University of Malaya, 50603 Kuala Lumpur, Malaysia

***=Corresponding authors**

Siew Woh Choo

Email: lawrence.choo@xjtlu.edu.cn

Nicholas S. Jakubovics

Tel: +44 (0) 191 208 6796 ; Email: nick.jakubovics@ncl.ac.uk

S1 Table: Functional enrichment analyses shows Ss unique core genes enriched in compound biosynthetic process (8) and cobalamin biosynthesis process (20).

Enriched biological process	Rast_ID	Enriched *Streptococcus sanguinis* unique core genes
Porphyrin-containing compound biosynthetic process	SK36.peg.479	Glutamate-1-semialdehyde aminotransferase (EC 5.4.3.8)
	SK36.peg.476	Porphobilinogen deaminase (EC 2.5.1.61)
	SK36.peg.475	Glutamyl-tRNA reductase (EC 1.2.1.70)
	SK36.peg.474	Siroheme synthase / Precorrin-2 oxidase (EC 1.3.1.76)
	SK36.peg.465	Uroporphyrinogen-III methyltransferase (EC 2.1.1.107) / Uroporphyrinogen-III synthase (EC 4.2.1.75)
	SK36.peg.461	Cobalt-precorrin-4 C11-methyltransferase (EC 2.1.1.133) /cobM
	SK36.peg.2040	FIG01117915: hypothetical protein
	SK36.peg.2038	FIG01118726: hypothetical protein
Cobalamin biosynthetic process	SK36.peg.481	Cobalamin synthase
	SK36.peg.480	Adenosylcobinamide-phosphate guanylyltransferase (EC 2.7.7.62) / cobU
	SK36.peg.472	Cobyric acid synthase
	SK36.peg.502	Nicotinate-nucleotide--dimethylbenzimidazole phosphoribosyltransferase (EC 2.4.2.21) / cobT
	SK36.peg.469	Additional substrate-specific component CbiN of cobalt ECF transporter
	SK36.peg.500	L-threonine 3-O-phosphate decarboxylase (EC 4.1.1.81)
	SK36.peg.468	Substrate-specific component CbiM of cobalt ECF transporter
	SK36.peg.467	Cobalt-precorrin-2 C20-methyltransferase (EC 2.1.1.130)
	SK36.peg.466	Sirohydrochlorin cobaltochelatase CbiK (EC 4.99.1.3)
	SK36.peg.464	Cobalt-precorrin-6x reductase (EC 1.3.1.54)
	SK36.peg.463	Cobalt-precorrin-3b C17-methyltransferase /cbiH
	SK36.peg.462	Cobalamin biosynthesis protein CbiG
	SK36.peg.461	Cobalt-precorrin-4 C11-methyltransferase (EC 2.1.1.133)
	SK36.peg.460	Cobalt-precorrin-6y C15-methyltransferase [decarboxylating] (EC 2.1.1.-)
	SK36.peg.459	Cobalt-precorrin-6y C5-methyltransferase (EC 2.1.1.-)
	SK36.peg.458	Cobalt-precorrin-6 synthase, anaerobic
	SK36.peg.457	Cobalt-precorrin-8x methylmutase (EC 5.4.1.2)
	SK36.peg.456	Cobalt-precorrin-8x methylmutase (EC 5.4.1.2)
	SK36.peg.455	Adenosylcobinamide-phosphate synthase /cbiP
	SK36.peg.454	Cobyrinic acid A, C-diamide synthase /cobB/cbiA

S2 Table: Overview of putative prophages including the size of the prophage, the number of CDS, ATT-site status and GC content.

Prophages	Length (kb)	CDS	ATT-site identified	GC content
7863_1	5.8kb	6	No	40.99%
FSS8_1	43.2kb	58	Yes	38.80%
SK12_1	36.5kb	54	No	41.25%
SK184_1	59.2kb	57	Yes	41.08%
SK184_3	48.7kb	75	Yes	40.74%
Channon_2	39.4Kb	62	Yes	38.76%
FSS4_1	30.9Kb	25	Yes	43.52%
FSS4_2	16.4Kb	29	Yes	40.31%
MB451_1	23.3Kb	26	Yes	43.51%
SK184_2	36kb	21	Yes	37.94%
SK184_4	6.9kb	11	No	43.49%
MB666_1	47Kb	32	Yes	40.29%

S3 Table: Overview of the putative genomic island (GI) details including the size of the GI, the number of CDS, GC contents and key genes incorporated in each GI.

GI	Size (bp)	Number of CDSs	GC content	Key Genes
GI_5	5253	5	33.70%	DNA recombination and repair protein RecF; FIG001621: Zinc protease; FIG009210: peptidase, M16 family and Transcriptional regulator in cluster with unspecified monosaccharide ABC transport system
GI_14	10312	6	20.50%	hypothetical proteins
GI_16	5085	6	38.90%	FIG007079: UPF0348 protein family; FIG145533: Methyltransferase (EC 2.1.1.-); Iojap protein;Hydrolase (HAD superfamily), YqeK and Nicotinate-nucleotide adenylyltransferase (EC 2.7.7.18)
GI_31	5557	6	33.60%	Permease of the drug/metabolite transporter (DMT) superfamily; TetR/AcrR family transcriptional regulator
GI_43	7035	10	42.20%	Integrase; Chromosome segregation helicase and MutT/nudix family protein; 7,8-dihydro-8-oxoguanine-triphosphatase
GI_45	5556	8	33.60%	Chromosome (plasmid) partitioning protein ParB / Stage 0 sporulation protein J; Serine protease, DegP/HtrA, do-like (EC 3.4.21.-); LSU m3Psi1915 methyltransferase RlmH and Competence pheromone precursor
GI_47	7627	12	43.20%	Integrase; Chromosome segregation helicase; MutT/nudix family protein; 7,8-dihydro-8-oxoguanine-triphosphatase; acetyltransferase,GNAT family;Ribosomal protein L11 methyltransferase (EC 2.1.1.-); Ribosomal RNA small subunit methyltransferase E (EC 2.1.1.-), and Mobile element protein (2 units)
GI_51	7355	9	31.90%	Chromosome (plasmid) partitioning protein ParB / Stage 0 sporulation protein J; Serine protease, DegP/HtrA, do-like (EC 3.4.21.-);LSU m3Psi1915 methyltransferase RlmH; Competence pheromone precursor; Histidine kinase of the competence regulon ComD; Response regulator of the competence regulon ComE; GTP-binding and nucleic acid-binding protein YchF; Peptidyl-tRNA hydrolase (EC 3.1.1.29) and Transcription-repair coupling factor
GI_53	4194	5	44.40%	CAAX amino terminal protease family family and Transcriptional regulator, TetR family
GI_55	5516	7	48.40%	V-type ATP synthase subunit C, E, F,G, I and K (EC 3.6.3.14) and Acetyltransferase, GNAT family
GI_58	7364	8	32.10%	Chromosome (plasmid) partitioning protein ParB / Stage 0 sporulation protein J; Serine protease, DegP/HtrA, do-like (EC 3.4.21.-); LSU m3Psi1915 methyltransferase RlmH; Competence pheromone precursor; Histidine kinase of the competence regulon ComD; Response regulator of the competence regulon ComE; GTP-binding and nucleic acid-binding protein YchF and Peptidyl-tRNA hydrolase (EC 3.1.1.29)
GI_67	4094	5	41.90%	Topoisomerase IV subunit B (EC 5.99.1.-) and lipoprotein, putative
GI_75	4183	5	44.60%	CAAX amino protease and Transcriptional regulator, TetR family

S4 Table: The genome sequencing results of 19 isolated *Streptococcus* strains using Next Generation Sequencing Illumina Hiseq 2000 platform.

Strain Name	Yield (Mbases)	Number of Reads	Mean Quality Score (PF)
**PV40**	826	8264126	36.24
**NCTC 7863**	822	8222024	35.69
**Blackburn**	1180	11797640	36.55
**Channon**	695	6949680	36.49
**FSS2**	882	8823944	36.67
**FSS3**	944	9442900	37.11
**FSS4**	1294	12943882	36.6
**FSS8**	1010	10102148	36.69
**FSS9**	988	9877224	36.61
**M5**	678	6784012	36.43
**M99**	666	6657462	36.19
**MB451**	1127	11271462	36.59
**MB666**	1095	10949508	36.81
**MW10**	1069	10693318	36.94
**PJM8**	1054	10543052	36.63
**PK488**	624	6240768	36.14
**SK12**	878	8782388	36.81
**SK120**	732	7324194	36.96
**SK184**	680	6795252	36.36

S5 Table: The sequencing coverage of 19 isolated *Streptococcus* strains based on the reference genome sizes of Sg Challis (2.2Mb) and Ss SK36 (2.39Mb).

Strain Name	Total Read Length (bp)	Genome Size (bp)	Sequencing Coverage
**PV40**	826412600	2200000	375.64
**Blackburn**	721326800	2200000	327.88
**Channon**	694968000	2200000	315.89
**FSS2**	882394400	2200000	401.09
**FSS3**	944290000	2200000	429.22
**FSS8**	1010214800	2200000	459.19
**M5**	678401200	2200000	308.36
**M99**	665746200	2200000	302.61
**MB666**	1094950800	2200000	497.70
**MW10**	1069331800	2200000	486.06
**NCTC7863**	822202400	2390000	344.02
**FSS4**	1294388200	2390000	541.59
**FSS9**	987722400	2390000	413.27
**MB451**	1127146200	2390000	471.61
**PJM8**	1054305200	2390000	441.13
**PK488**	624076800	2200000	283.67
**SK12**	878238800	2200000	399.20
**SK120**	732419400	2200000	332.92
**SK184**	679525200	2200000	308.88

S1 Figure. The heatmap generated for comparative pathogenomics analysis between 15 Sg strains and 27 Ss strains using a threshold of 50% sequence identity and 50% sequence coverage.

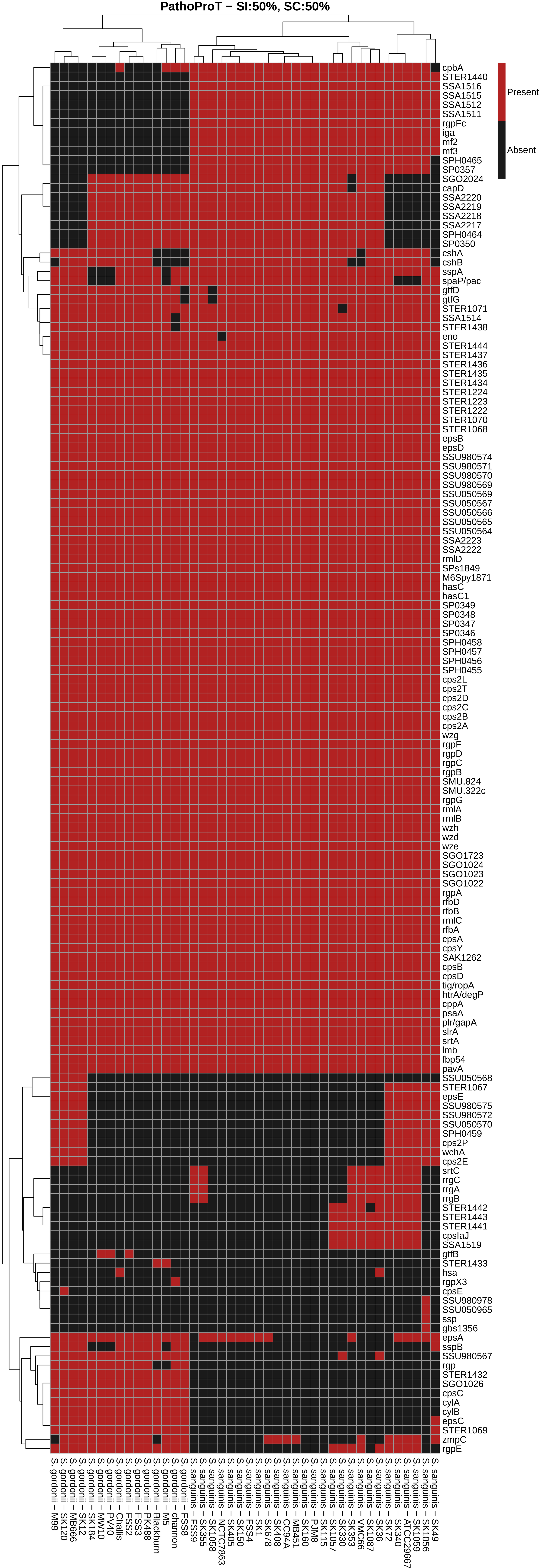

PathoProT − SI:50%, SC:50%
Present
Absent
cpbA
STER1440
SSA1516
SSA1515
SSA1512
SSA1511
rgpFc
iga
mf2
mf3
SPH0465
SP0357
SGO2024
capD
SSA2220
SSA2219
SSA2218
SSA2217
SPH0464
SP0350
cshA
cshB
sspA
spaP/pac
gtfD
gtfG
STER1071
SSA1514
STER1438
eno
STER1444
STER1437
STER1436
STER1435
STER1434
STER1224
STER1223
STER1222
STER1070
STER1068
epsB
epsD
SSU980574
SSU980571
SSU980570
SSU980569
SSU050569
SSU050567
SSU050566
SSU050565
SSU050564
SSA2223
SSA2222
rmlD
SPs1849
M6Spy1871
hasC
hasC1
SP0349
SP0348
SP0347
SP0346
SPH0458
SPH0457
SPH0456
SPH0455
cps2L
cps2T
cps2D
cps2C
cps2B
cps2A
wzg
rgpF
rgpD
rgpC
rgpB
SMU.824
SMU.322c
rgpG
rmlA
rmlB
wzh
wzd
wze
SGO1723
SGO1024
SGO1023
SGO1022
rgpA
rfbD
rfbB
rmlC
rfbA
cpsA
cpsY
SAK1262
cpsB
cpsD
tig/ropA
htrA/degP
cppA
psaA
plr/gapA
slrA
srtA
lmb
fbp54
pavA
SSU050568
STER1067
epsE
SSU980575
SSU980572
SSU050570
SPH0459
cps2P
wchA
cps2E
srtC
rrgC
rrgA
rrgB
STER1442
STER1443
STER1441
cpslaJ
SSA1519
gtfB
STER1433
hsa
rgpX3
cpsE
SSU980978
SSU050965
ssp
gbs1356
epsA
sspB
SSU980567
rgp
STER1432
SGO1026
cpsC
cylA
cylB
epsC
STER1069
zmpC
rgpE
S. gordonii − M99
S. gordonii − SK120
S. gordonii − MB666
S. gordonii − SK12
S. gordonii − SK184
S. gordonii − MW10
S. gordonii − PV40
S. gordonii − Challis
S. gordonii − FSS2
S. gordonii − FSS3
S. gordonii − PK488
S. gordonii − Blackburn
S. gordonii − M5
S. gordonii − channon
S. gordonii − FSS8
S. sanguinis − FSS9
S. sanguinis − SK355
S. sanguinis − SK1058
S. sanguinis − NCTC7863
S. sanguinis − SK405
S. sanguinis − SK150
S. sanguinis − FSS4
S. sanguinis − SK1
S. sanguinis − SK678
S. sanguinis − SK408
S. sanguinis − CC94A
S. sanguinis − MB451
S. sanguinis − SK160
S. sanguinis − PJM8
S. sanguinis − SK115
S. sanguinis − SK1057
S. sanguinis − SK330
S. sanguinis − SK353
S. sanguinis − VMC66
S. sanguinis − SK1087
S. sanguinis − SK36
S. sanguinis − SK72
S. sanguinis − SK340
S. sanguinis − ATCC29667
S. sanguinis − SK1059
S. sanguinis − SK1056
S. sanguinis − SK49

S2 Figure. The visualization of Sg Challis-type polysaccharide gene cluster structure in Sg and Ss using Mauve software.

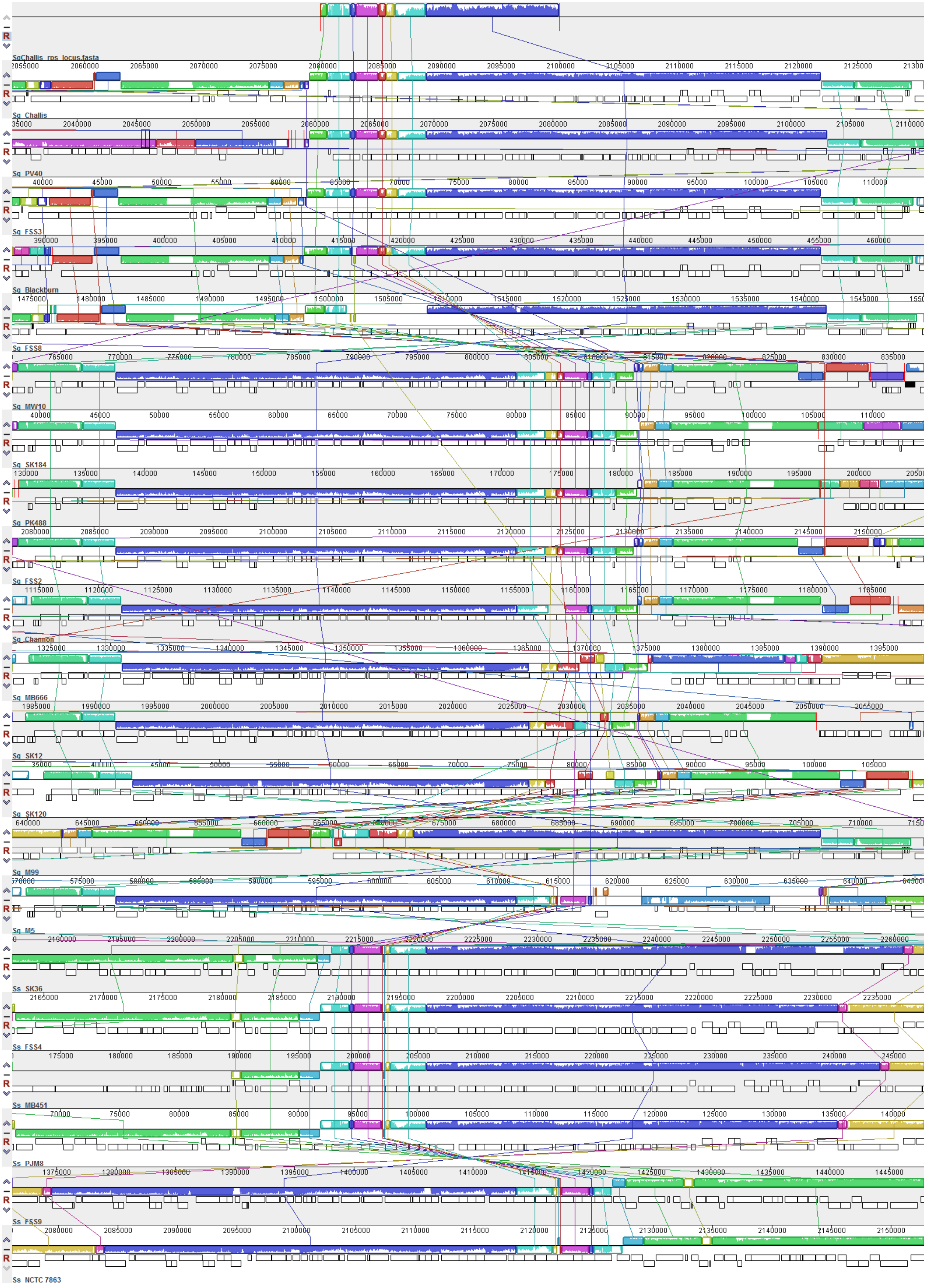

S3 Figure. A. Adhesion of Sg M99, PK488 and DL1 to fetuin (red bars) or sialidase-treated fetuin (yellow bars). The substrate was immobilised on a plastic surface and exposed to bacteria for 2 h at 37°C. After washing, bound bacterial cells were stained with crystal violet and quantified by measuring A_{595} as described previously[1]. Bars represent means from three independent experiments and standard errors are shown. B. Phylogenetic analysis of N-terminal binding regions of serine-rich region proteins from Ss and Sg. The evolutionary history was inferred using the Neighbor-Joining method using *S. pneumoniae* INV200 PsrP as an outgroup[2]. The percentage of replicate trees in which the associated taxa clustered together in the bootstrap test (500 replicates) are shown next to the branches[3]. The tree is drawn to scale, with branch lengths in the same units as those of the evolutionary distances used to infer the phylogenetic tree. The evolutionary distances were computed using the Poisson correction method[4] and are shown as the number of amino acid substitutions per site. Evolutionary analyses were conducted in MEGA7[5]. The binding region sequences of Sg PK488 and FSS4 clustered separately from the relatively tight GspB and Hsa variant groups (Sg strains) or the SrpA-type proteins, which were found only in Ss strains.

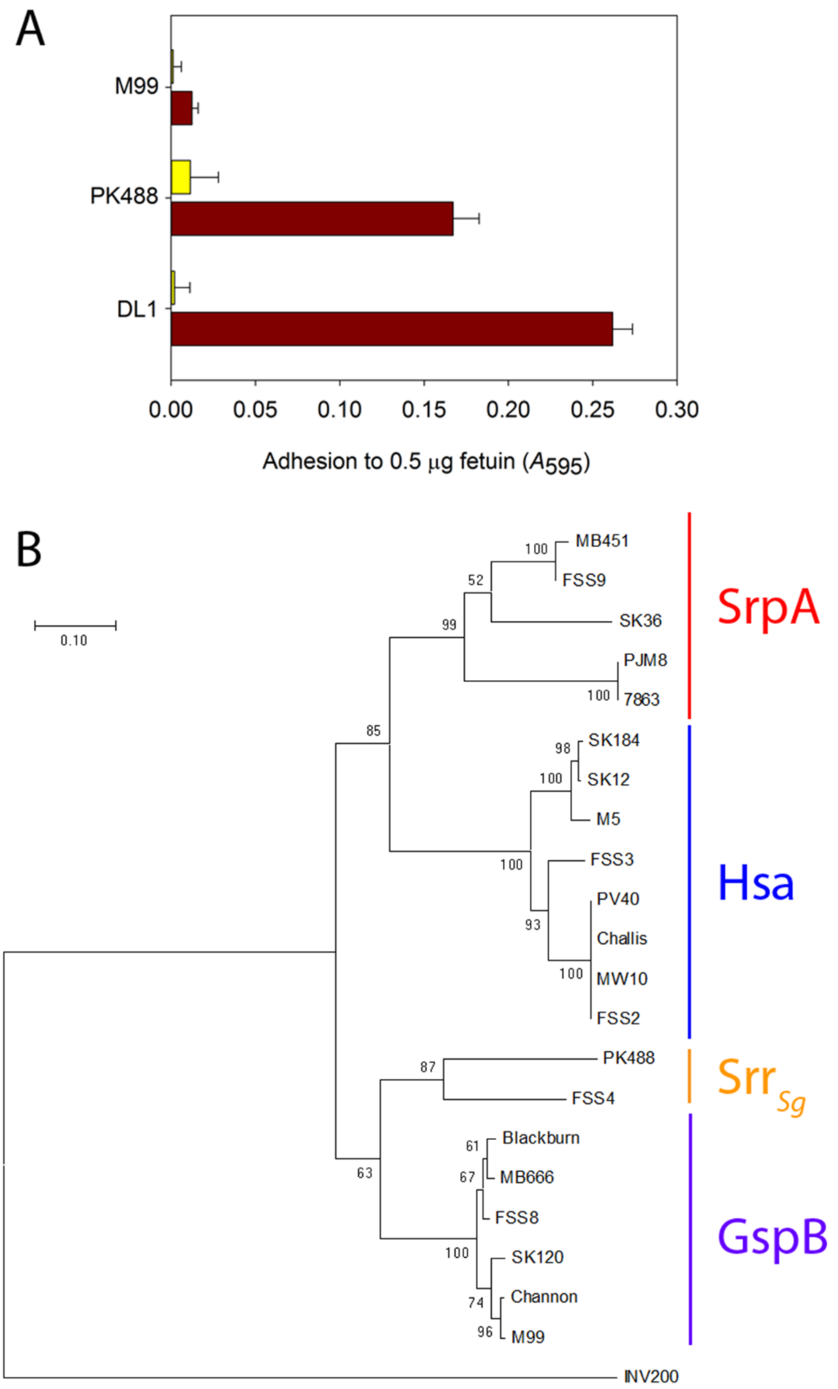

Supplemental references

1. Jakubovics, N.S., Brittan, J. L., Dutton, L.C. & Jenkinson, H.F. Multiple adhesin proteins on the cell surface of *Streptococcus gordonii* are involved in adhesion to human fibronectin. *Microbiology* **155**, 3572-3580 (2009).

2. Saitou N. & Nei M. The neighbor-joining method: A new method for reconstructing phylogenetic trees. *Molecular Biology and Evolution* **4**, 406-425 (1987).

3. Felsenstein J. Confidence limits on phylogenies: An approach using the bootstrap. *Evolution* **39**, 783-791 (1985).

4. Zuckerkandl E. & Pauling L. Evolutionary divergence and convergence in proteins. Edited in Evolving Genes and Proteins by V. Bryson and H.J. Vogel, pp. 97-166. Academic Press, New York (1965).

5. Kumar S., Stecher G., & Tamura K. MEGA7: Molecular Evolutionary Genetics Analysis version 7.0 for bigger datasets. *Molecular Biology and Evolution* 33:1870-1874 (2016).
